# Systemic AAV6-synapsin-GFP administration results in lower liver biodistribution, compared to AAV1&2 and AAV9, with neuronal expression following ultrasound-mediated brain delivery

**DOI:** 10.1038/s41598-021-81046-5

**Published:** 2021-01-21

**Authors:** Danielle Weber-Adrian, Rikke Hahn Kofoed, Joseph Silburt, Zeinab Noroozian, Kairavi Shah, Alison Burgess, Shawna Rideout, Sebastian Kügler, Kullervo Hynynen, Isabelle Aubert

**Affiliations:** 1 Present Address: Faculty of Health Sciences, School of Medicine, Queen′s University, Kingston, ON Canada; 2 Biological Sciences, Hurvitz Brain Sciences Research Program, Sunnybrook Research Institute, Toronto, ON Canada; 3 Department of Laboratory Medicine and Pathobiology, Temerty Faculty of Medicine, University of Toronto, Toronto, ON Canada; 4 Institute of Medical Sciences, Temerty Faculty of Medicine, University of Toronto, Toronto, ON Canada; 5 Physical Sciences, Sunnybrook Research Institute, Toronto, ON Canada; 6 Department of Neurology, Center Nanoscale Microscopy and Physiology of the Brain (CNMPB) at University Medical Center Göttingen, Göttingen, Germany; 7 Department of Medical Biophysics, Temerty Faculty of Medicine, University of Toronto, Toronto, ON Canada

**Keywords:** Blood-brain barrier, Molecular neuroscience

## Abstract

Non-surgical gene delivery to the brain can be achieved following intravenous injection of viral vectors coupled with transcranial MRI-guided focused ultrasound (MRIgFUS) to temporarily and locally permeabilize the blood–brain barrier. Vector and promoter selection can provide neuronal expression in the brain, while limiting biodistribution and expression in peripheral organs. To date, the biodistribution of adeno-associated viruses (AAVs) within peripheral organs had not been quantified following intravenous injection and MRIgFUS delivery to the brain. We evaluated the quantity of viral DNA from the serotypes AAV9, AAV6, and a mosaic AAV1&2, expressing green fluorescent protein (GFP) under the neuron-specific synapsin promoter (syn). AAVs were administered intravenously during MRIgFUS targeting to the striatum and hippocampus in mice. The syn promoter led to undetectable levels of GFP expression in peripheral organs. In the liver, the biodistribution of AAV9 and AAV1&2 was 12.9- and 4.4-fold higher, respectively, compared to AAV6. The percentage of GFP-positive neurons in the FUS-targeted areas of the brain was comparable for AAV6-syn-GFP and AAV1&2-syn-GFP. In summary, MRIgFUS-mediated gene delivery with AAV6-syn-GFP had lower off-target biodistribution in the liver compared to AAV9 and AAV1&2, while providing neuronal GFP expression in the striatum and hippocampus.

## Introduction

Gene therapy for the treatment of neurodegenerative disorders, such as spinal muscular atrophy^1^, Alzheimer’s^2^ and Parkinson’s^3^ diseases, has reached the clinical trial stage using adeno-associated virus (AAV) as a gene expression vector. However, non-invasive and brain-specific gene delivery remains a challenge. Most AAV serotypes cannot overcome the blood–brain barrier (BBB) and, to date, intracranial surgeries are required for targeted gene delivery the brain. AAV serotype 9 (AAV9) has the capacity to cross the BBB when administered intravenously, at a minimum of 1 × 10^11^ vector genomes or viral particles per mouse^4,5^. Such systemic administration of AAV9 results in non-targeted central and peripheral transduction and expression.

MRI-guided focused ultrasound (MRIgFUS), in the presence of microbubbles, triggers a localized and transient BBB permeabilization^6^. Thereby, MRIgFUS offers a promising method to mediate non-surgical gene delivery to targeted brain areas using AAV serotypes which do not overcome the BBB^7^, and AAV9 at dosages below the systemic minimum required to pass the BBB^8–10^. Targeted AAV9 delivery to the brain using MRIgFUS has been previously demonstrated^8^. However, systemic injection of AAV9 expressing green fluorescent protein (GFP) also resulted in substantial transgene expression in the liver^8^. Since AAV transduction has been shown to initiate cytotoxic T cell-mediated destruction of hepatocytes^11^, this prompted the current investigation of serotypes to limit AAV DNA biodistribution in the liver, combined with a promoter that would prevent transgene transcription.

In terms of serotypes which do not overcome the BBB, AAV2 is one of the best characterized serotypes and, to date, the most used in clinical trials^12^, while AAV1 has demonstrated greater expression efficiency in the brain compared to AAV2^13–15^. The mosaic AAV1&2 combination serotype shows the neuron-affinity of AAV2 and the greater diffusion efficiency into the brain parenchyma of AAV1^16^, particularly in the striatum as compared to AAV2^17^. Additionally, AAV1&2 has demonstrated transgene expression in the brain after injection in the periphery of neonatal mice^18^. AAV6 also demonstrates affinity for neuronal tissue^19–22^, with better transduction in the central nervous system (CNS) compared to AAV1 and AAV2^15,23,24^, and reduced tropism for the liver compared to AAV9^4^. Using MRIgFUS delivery to the caudate putamen, a brain region implicated in Parkinson’s disease, a previous study tested GFP expression from AAV1 and AAV2 under control of the synapsin (syn) promoter, reporting mainly neuronal expression^25^. In peripheral organs, the syn promoter prevented transgene expression; however, the biodistribution of AAV DNA was not investigated^25^. Furthermore, brain regions of clinical relevance to Alzheimer’s disease^26^, such as the hippocampus, had yet to be characterized for MRIgFUS gene delivery using AAVs under the syn promoter. Here, a mosaic AAV1&2 serotype is compared with AAV6 under control of the syn promoter, with MRIgFUS-mediated delivery to the hippocampus and striatum. Biodistribution of transgene DNA within the liver, kidney, muscle, heart, spleen, and lung was quantified. Our results indicate that MRIgFUS AAV6-syn-GFP gene delivery leads to transgene expression below the detectable limit of our assay in the liver, lower DNA biodistribution within the liver compared to AAV1&2, and provides neuronal GFP expression in the hippocampus and striatum. The development of AAV serotypes and promoters for MRIgFUS gene therapy could tailor treatment to specific neurological disorders and brain regions, without being limited to serotypes which innately overcome the BBB or surgical delivery.

## Results

### AAV6 results in less biodistribution within the liver than AAV1&2 and AAV9

**Figure 1:**
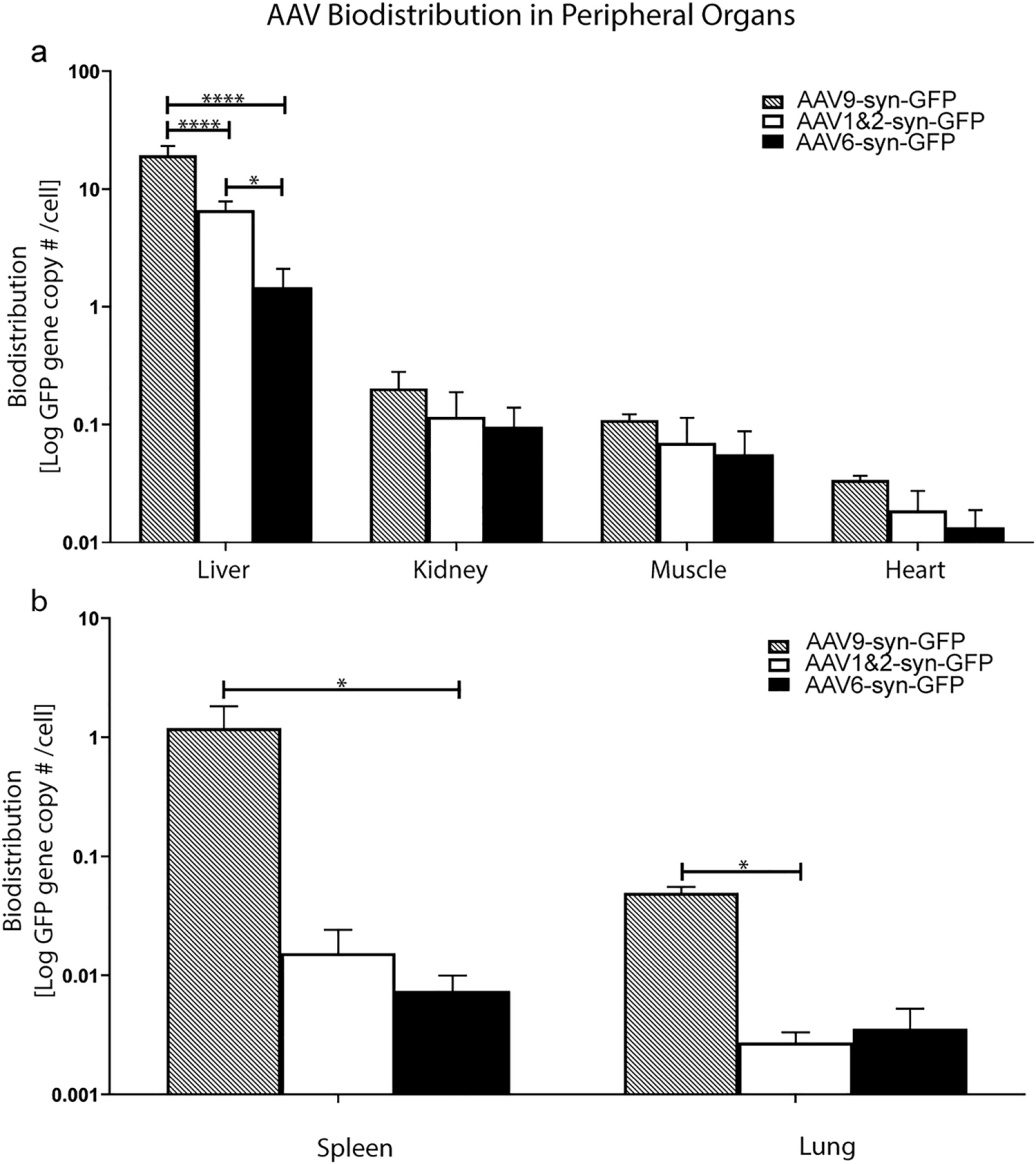
Transgene DNA biodistribution in peripheral organs. GFP gene copy number per cell within the liver, kidney, muscle, heart, spleen and lung was evaluated using droplet digital PCR. Systemic injection of AAV9-syn-GFP resulted in 2.9-fold greater biodistribution in the liver compared to AAV1&2 (*p* < 0.0001), and 12.9-fold greater biodistribution than AAV6 (*p* < 0.0001). Delivery of AAV1&2-syn-GFP resulted in a 4.4-fold greater biodistribution in the liver (*p < 0.05) compared to AAV6-syn-GFP. The liver demonstrated the highest rate of biodistribution compared to the other organs. There was no significant difference in biodistribution efficiency between AAV9, AAV1&2 and AAV6 in the kidney, muscle or heart (**a**). AAV biodistribution in the spleen and lung occurred at a much smaller scale compared to the liver. AAV9 biodistribution was significantly higher in the spleen compared to that of AAV6 (*p < 0.05). In the lung, AAV9 biodistribution was significantly higher than that of AAV1&2 (*p < 0.05) (**b**). Data is represented as the mean ± SEM, with n = 3 animals per group.

Biodistribution of AAV-syn-GFP DNA was quantified as GFP gene copy numbers per cell using droplet digital PCR (ddPCR) in the liver, kidney, muscle, heart, spleen and lung. Three AAV serotypes were compared after systemic injection of 3 × 10^9^ vector genomes per gram (VG/g) in C57BL/6 mice: specifically, AAV9, AAV1&2 and AAV6. AAV9 injection resulted in 2.9-fold greater biodistribution in the liver compared to AAV1&2 (*p* < 0.0001), and 12.9-fold greater biodistribution compared to AAV6 (*p* < 0.0001). In turn, AAV1&2 had 4.4-fold greater biodistribution within the liver compared to AAV6 (*p* = 0.01, Fig. 1a). The next highest levels of biodistribution were measured in the kidney, which were an order of magnitude less than the liver for AAV1&2 and AAV6, and two orders of magnitude less for AAV9. There was no significant difference between biodistributions of AAV9, AAV1&2 and AAV6 in the kidney, muscle or heart (listed in order of decreasing biodistribution, Fig. 1a). In the spleen, AAV9 delivery resulted in significantly higher biodistribution than AAV6 (*p* = 0.01), but not AAV1&2 (*p* = 0.08) (Fig. 1b). In the lung, AAV9 showed levels of biodistribution that were significantly higher than AAV1&2 (*p* = 0.01), but not AAV6 (*p* = 0.27) (Fig. 1b).

**Figure 2:**
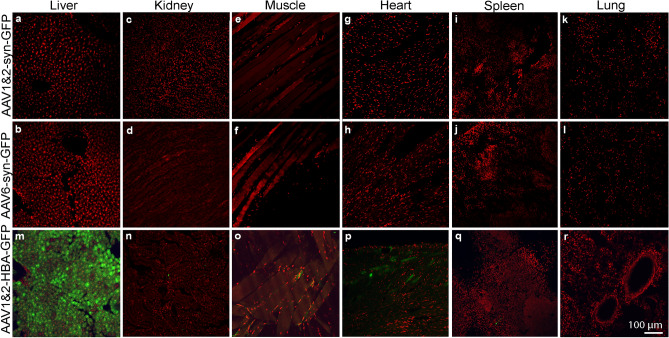
No detection of transgene expression in peripheral organs under syn promoter control. Intravenous administration of AAV1&2-syn-GFP and AAV6-syn-GFP resulted in no transgene expression in the liver (**a**, **b**), kidney (**c**, **d**), muscle (**e**, **f**), heart (**g**, **h**), spleen (**I**, **j**), and lung (**k**, **l**). Gene expression (GFP, green) after delivery of AAV1&2 under control of the human beta actin (HBA) promoter was conversely detected in the liver (**m**), kidney (**n**), muscle (**o**), heart (*p*), and spleen (**q**). No GFP expression was detected in the lung after injection of either AAV1&2-syn-GFP, AAV6-syn-GFP, or AAV1&2-HBA-GFP. Nuclear staining is shown with DAPI (red).

Under control of the synapsin promoter, neither AAV1&2-syn-GFP nor AAV6-syn-GFP delivery resulted in detectable GFP expression in the peripheral organs (DAPI, red), even when an anti-GFP antibody was used to enhance signal (Fig. 2a–l). Conversely, expression of GFP after delivery of AAV1&2-HBA-GFP (HBA-human beta actin promoter) was visible in the liver, kidney, muscle, heart, and spleen, but was undetected in the lung (Fig. 2m–r).

### AAV6-syn-GFP results in a comparable number of GFP-expressing neurons in FUS targeted brain regions compared to AAV1&2-syn-GFP

**Figure 3:**
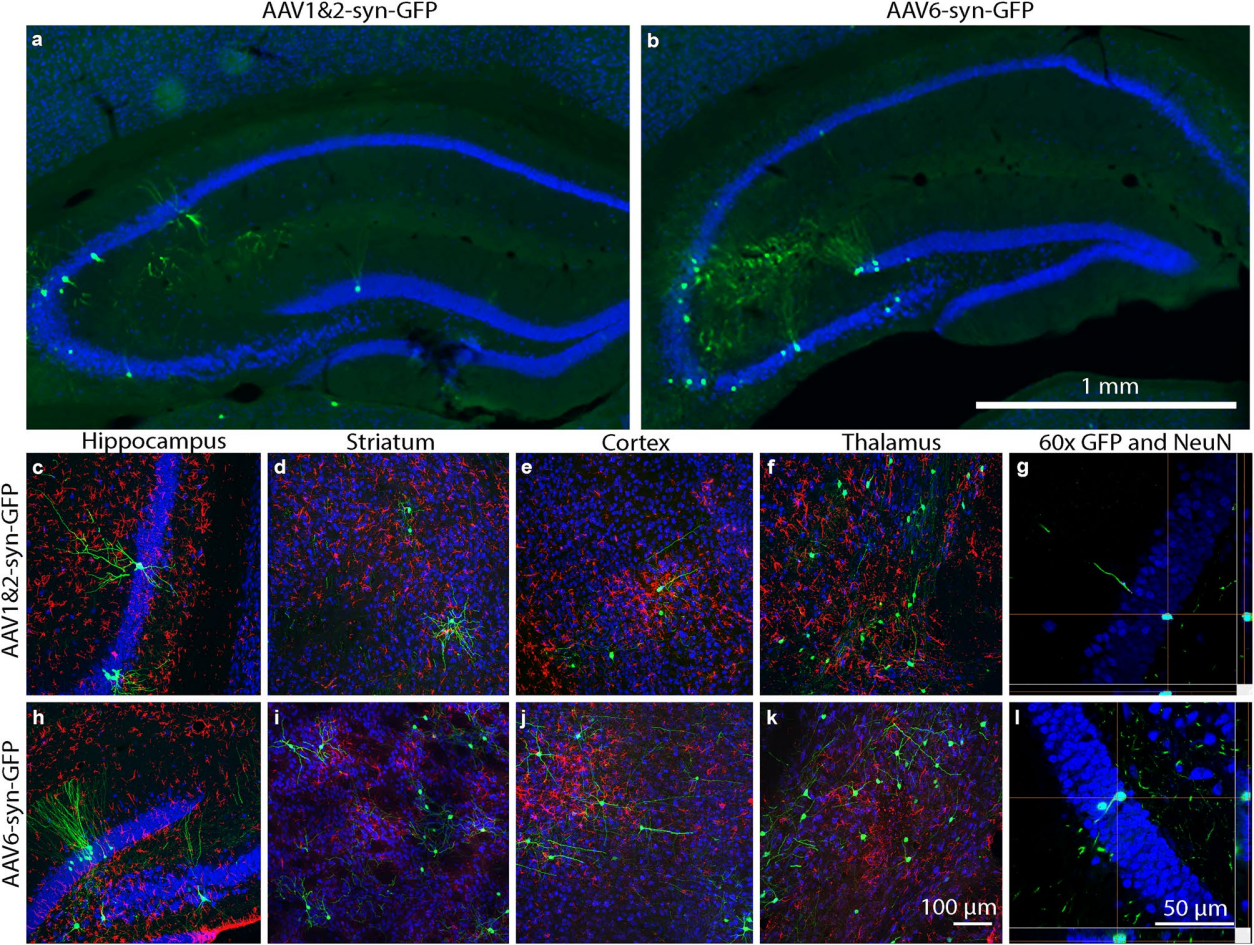
Transgene expression in neurons after MRIgFUS delivery of AAV to the hippocampus, striatum, thalamus, and cortex. AAV1&2-synapsin-green fluorescent protein (AAV1&2-syn-GFP) (**a**, **c–g**) and AAV6-syn-GFP (**b**, **h–l**) were delivered from the blood to the brain, unilaterally, via MRIgFUS permeabilization of the bood-brain barrier. A 20 × compiled virtual slice of the hippocampus shows GFP-positive neurons (GFP, green; NeuN, blue) after delivery of AAV1&2 and AAV6 (**a**, **b**). AAV1&2 and AAV6 resulted in GFP expression (GFP, green; NeuN, blue; GFAP, red) in the hippocampus (**c**, **h**), striatum (**d**, **i**), cortex (**e**, **j**), and thalamus (**f**, **k**). At 60 × magnification (**g and l**), colocalization between neuronal cell type (NeuN, blue) and transgene expression (GFP, green) is shown in turquoise.

MRIgFUS delivery of AAV1&2-syn-GFP and AAV6-syn-GFP from the blood (3 × 10^9^ VG/g) to the hippocampus, striatum, thalamus, and cortex resulted in GFP transgene expression (Fig. 3). GFP expression is evident in the hippocampus of both AAV1&2-syn-GFP and AAV6-syn-GFP-injected animals (Fig. 3a,b). At higher magnification, GFP expression can be seen throughout the cell body and processes in the hippocampus (Fig. 3c,h), striatum (Fig. 3d,i), cortex (Fig. 3e,j), and thalamus (Fig. 3f,k). GFP-positive cell bodies were exclusively co-localized with NeuN-positive (neuronal marker) cells (Fig. 3g,l). This confirms neuron-specific transgene expression in the brain under the syn promoter in conjunction with the AAV1&2 and 6 serotypes.

**Figure 4:**
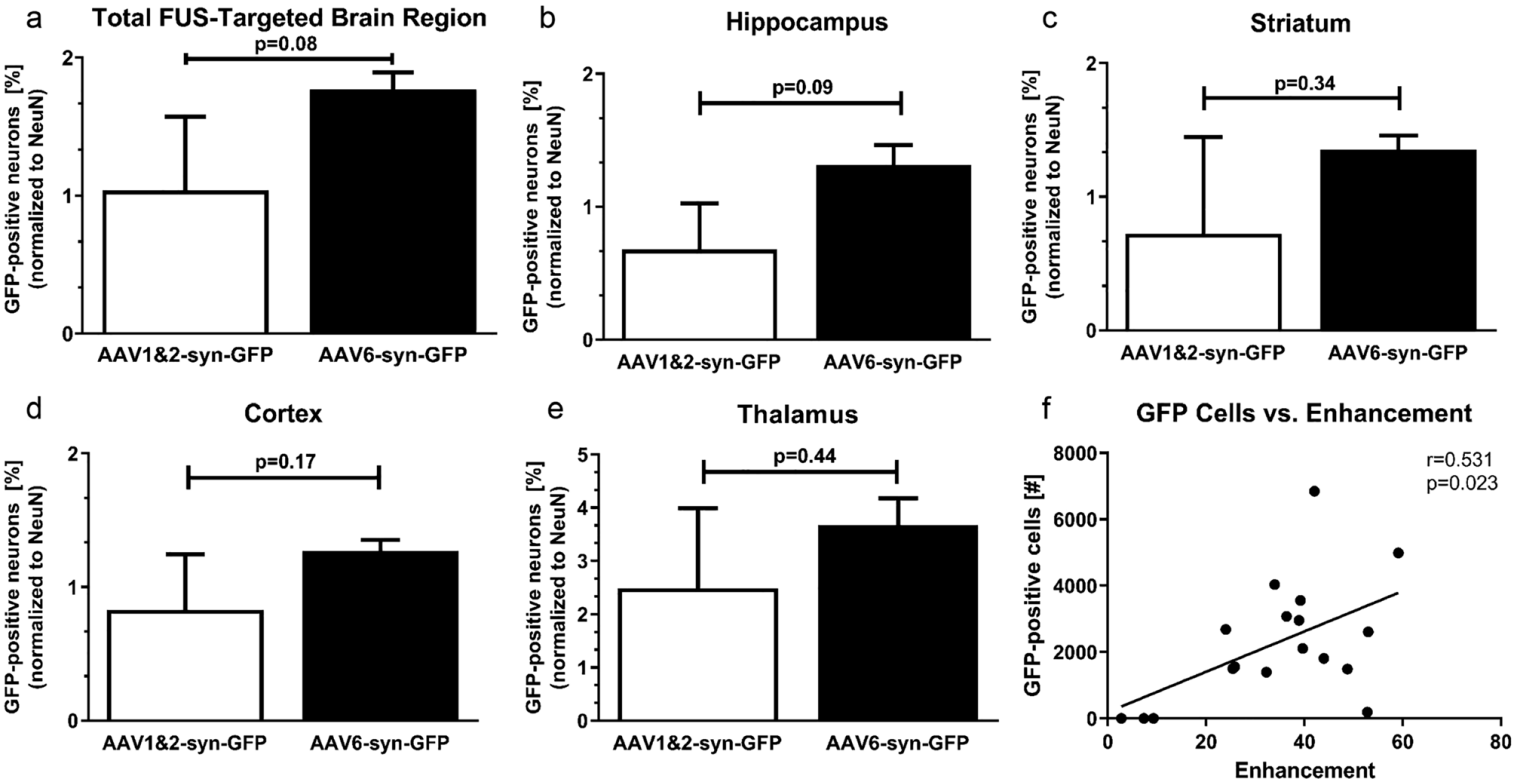
Quantification of neuronal transgene expression following MRIgFUS delivery of AAV1&2 and AAV6 to the brain. Focused ultrasound (FUS) delivery of AAV6-syn-GFP did not result in a statistically significant increase in percentage of GFP-positive neurons compared to AAV1&2-syn-GFP in the total GFP-positive region of the brain (*p* = 0.08) (**a**) and hippocampus (*p* = 0.09) (**b**)**.** There was also no significant difference in percentage of GFP-positive neurons in the striatum (*p* = 0.34) (**c**), cortex (*p* = 0.17) (**d**), or thalamus (*p* = 0.44) (**e**). Data is represented as the mean ± SEM, percent of co-labelled GFP and neuronal nuclei antigen (NeuN)-positive cells from the total NeuN-positive population. For quantification, 3 distinct, non-overlapping FUS focal spots per animal were averaged for each reported percentage, where n = 3 animals per group. An ANCOVA was used to isolate the effect of serotype on percentage of GFP-positive cells since a weak (r = 0.531), but significant (*p = 0.023) correlation was found between the number of GFP-positive cells within the region of FUS application and MRI enhancement (n = 18 focal points) (**f**).

AAV6-syn-GFP delivery did not result in a statistically significant difference in percentage of GFP and NeuN-positive cells within the FUS-targeted area compared to AAV1&2-syn-GFP (Fig. 4a, p = 0.08, n = 3 animals per group). Power analyses revealed that sample sizes of 14 mice per AAV group would have an 80% power to detect differences between means of AAV1/2 and AAV6 GFP-positive cells in the total brain (Fig. 4a) with a significance level (alpha) of 0.05 (two-tailed). Percentages were reported from the total NeuN-positive cell population in the same area. There was also no significant difference in percentage of GFP-positive cells within the hippocampus (*p* = 0.09), striatum (*p* = 0.34), cortex (*p* = 0.17) or thalamus (*p* = 0.44) specifically after AAV6-syn-GFP delivery compared to AAV1&2-syn-GFP (Fig. 4b–e).

### MRI enhancement is comparable between experimental groups

**Figure 5:**
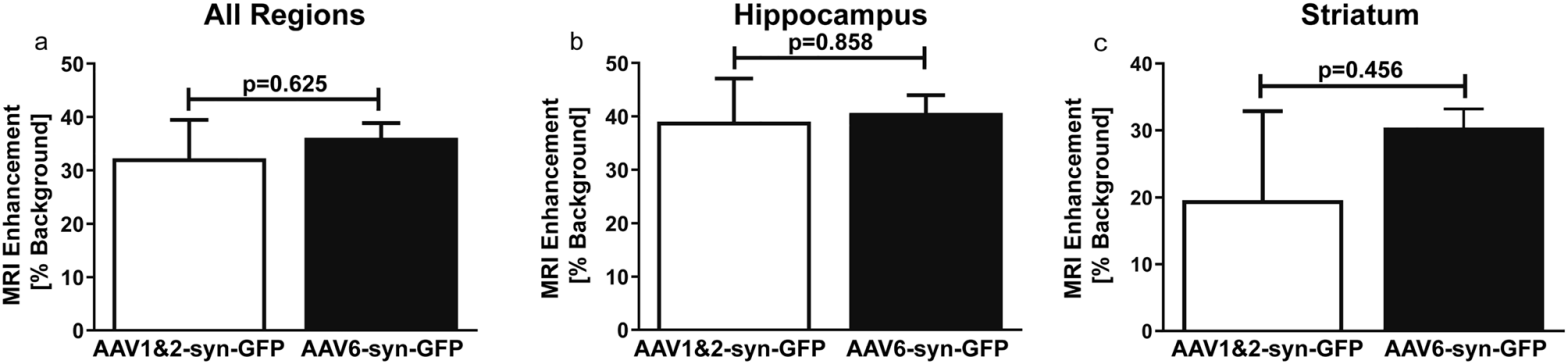
Blood–brain barrier opening is not significantly different between adeno-associated virus (AAV) groups. The mean MRI enhancements of the focal spots generated by FUS were not significantly different between the AAV6-syn-GFP and AAV1&2-syn-GFP experimental groups (**a**)**.** There was also no difference detected in enhancement of the focal spots targeting the hippocampus specifically (**b**), nor the striatum (**c**)**.** MRI enhancement is expressed as percent increase intensity over background. Data is represented as the mean ± SEM, n = 9 focal spots per group (**a**), n = 5 per group for the hippocampus (**b**) , n = 4 per group for the striatum (**c**).

The mean MRI enhancement of FUS-targeted spots in the striatum and hippocampus were not significantly different (*p* = 0.625) between the groups receiving AAV1&2-syn-GFP or AAV6-syn-GFP (Fig. 5a). There was also no significant difference in the mean enhancement of the focal spots when separated into those targeting regions of the hippocampus (*p* = 0.986) (Fig. 5b) versus regions of the striatum (*p* = 0.456) (Fig. 5c). MRI enhancement is correlated with the number of GFP positive cells in each FUS focal spot (Fig. 4f). It was therefore necessary to compare the enhancement of focal spots in the AAV1&2 vs AAV6 groups to ensure that any potential differences were due to the viral serotypes and not FUS application.

### MRIgFUS-mediated AAV delivery results in targeted, unilateral transgene expression

**Figure 6:**
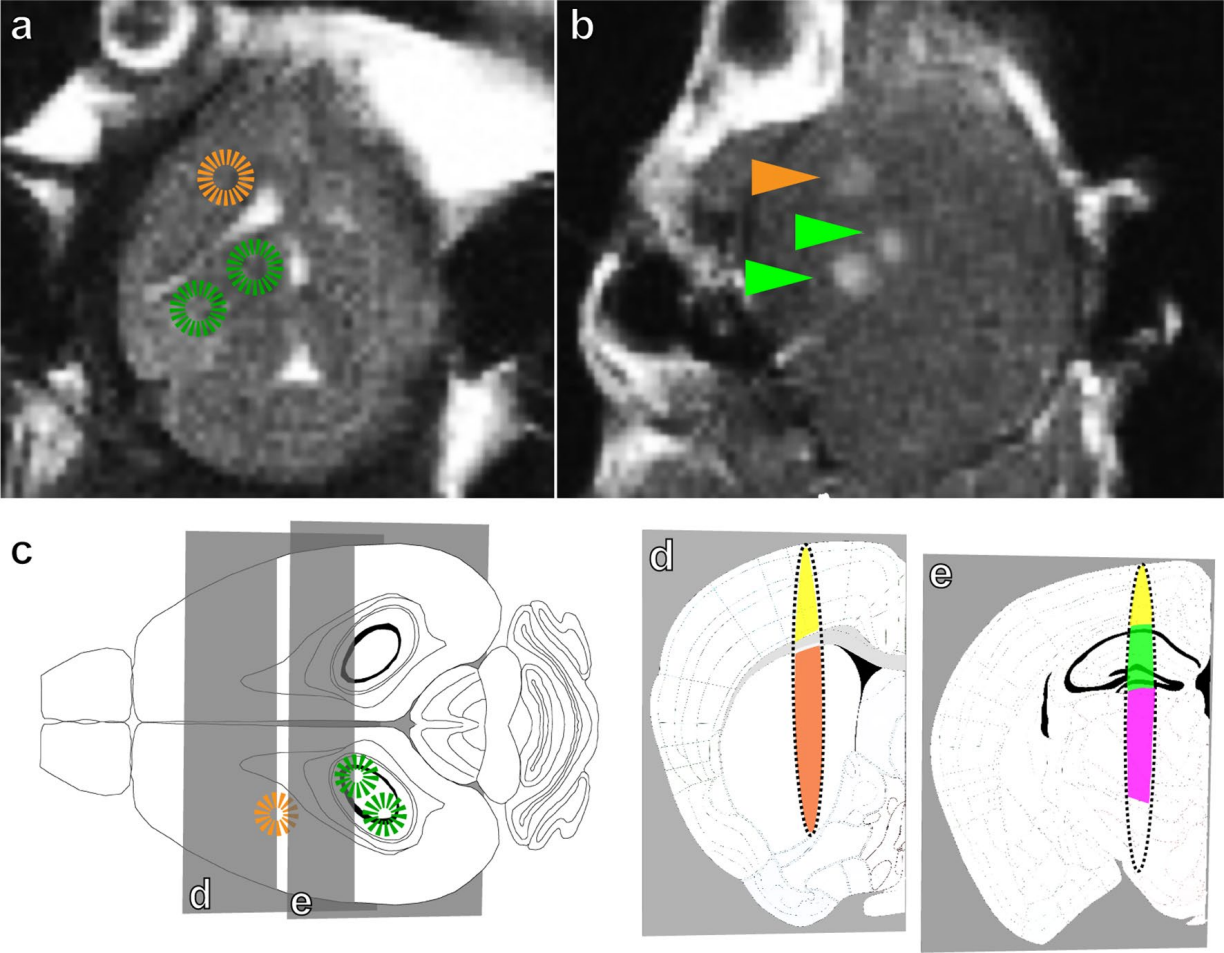
MRI-guided focused ultrasound (MRIgFUS)-mediated blood–brain barrier permeabilization. A T2-weighted MRI image of the brain was generated to target the desired areas with focused ultrasound (FUS). Targeting locations are indicated by circles (**a**). After FUS, a gadolinium-enhanced T1-weighted image was used to confirm blood–brain barrier disruption, as indicated by the regions of enhancement (arrowheads) in the targeted areas (**b**), namely the striatum (orange) and hippocampus medially and laterally (green) (**c**)**.** At these parameters, FUS targeting results in an oval focal spot, as illustrated in coronal views (**d**, **e**), which covers areas of the cortex (yellow), striatum (orange) (**d**), hippocampus (green) (**e**) , and thalamus (pink) (**e**). Figures 1d and e were adapted from the Allen Mouse Brain atlas (http://mouse.brain-map.org). Image credit: Allen Institute.

**Figure 7:**
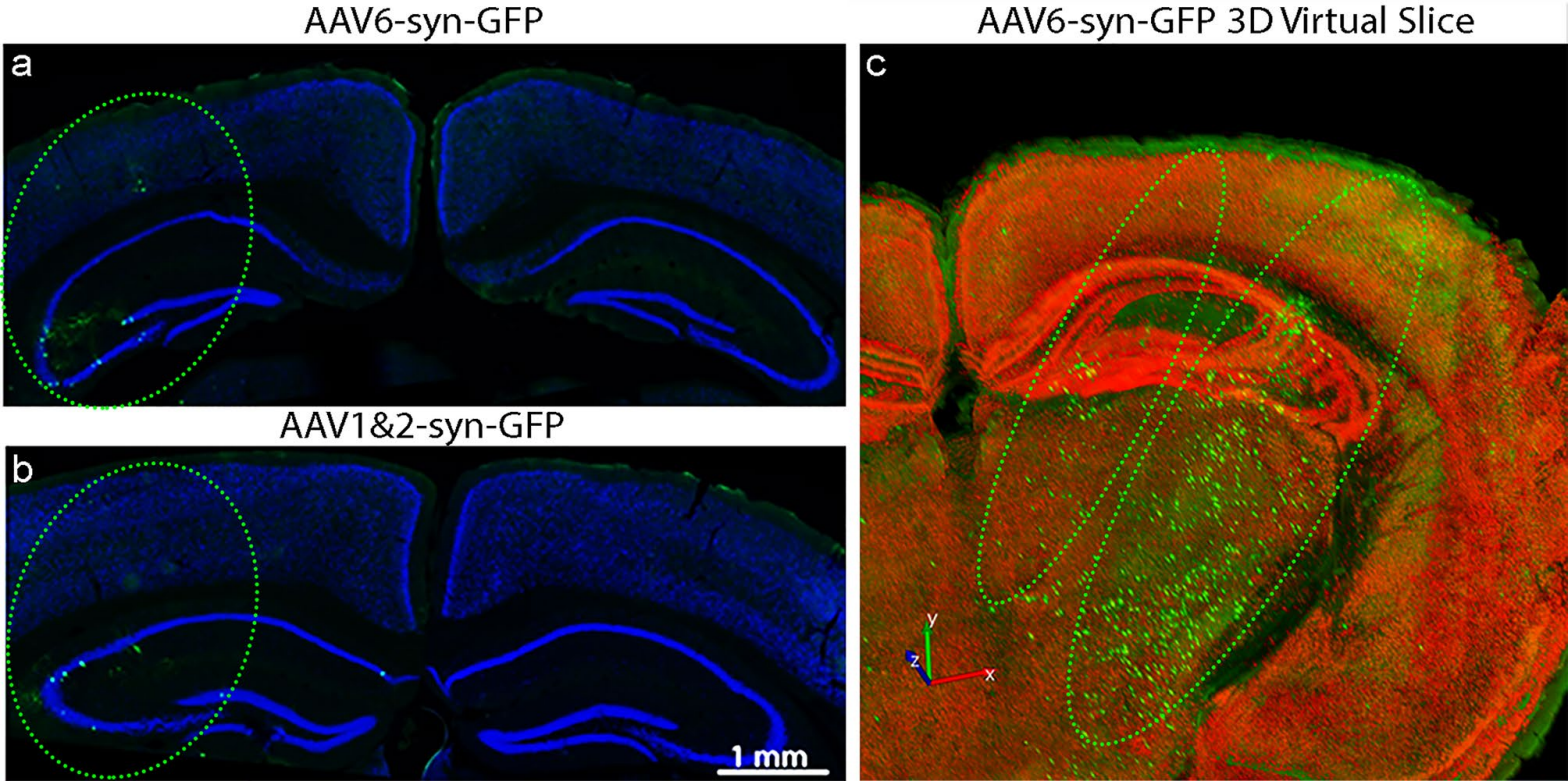
Unilateral AAV Transgene Delivery to the Brain Using MRI-Guided Focused Ultrasound. Virtual slice composite images of the cortex and hippocampus after AAV6-syn-GFP (**a**) and AAV1&2-syn-GFP (**b**) delivery demonstrate unilateral GFP expression (green ovals). A 3D composite of 10 virtual slice images from a representative AAV6-syn-GFP-treated animal is shown, with images optimized for one focal area targeting the lateral hippocampus (larger oval) (**c**). Accompanying GFP expression from targeting of the medial hippocampus in an adjacent FUS focal spot is also visible (small oval).

After unilateral FUS application to the striatum and hippocampus using three focal spots (Fig. 6), AAV1&2 and AAV6-syn-GFP expression is visible on only the targeted side of the brain (Fig. 7a,b). AAV1&2 and AAV6-syn-GFP did not lead to biodistribution or expression on the contralateral side of the brain. This supports that, without FUS, AAV1&2 and AAV6 do not cross the BBB. A 3D reconstruction optimized for one of two hippocampus-targeting FUS spots demonstrates a distribution pattern of transgene expression that is consistent with the size and shape of the focal spot generated using these FUS parameters (Fig. 7c).

## Discussion

We compared the efficiency and specificity of two non-BBB penetrating AAV serotypes, expressing GFP under the control of the neuron-specific syn promoter, delivered to the hippocampus and striatum from the bloodstream using MRIgFUS. Namely, the mosaic AAV1&2 and AAV6 were found to expressed GFP in the striatum, hippocampus, and also in areas located within the z-axis of the focal beam, i.e. the cortex and thalamus. AAV6-syn-GFP resulted in 4.4-fold less biodistribution in the liver, compared to the mosaic AAV1&2-syn-GFP, and the syn promoter prevented detectable transgene expression in peripheral organs examined, including the liver, after systemic delivery of AAV.

There has been increasing evidence that MRIgFUS can mediate efficient, targeted and non-surgical AAV delivery to the brain^7,8,10,25,27^. MRIgFUS may circumvent the risks associated with intracranial injection of AAV, which include tissue damage caused by the needle track^28^ and infection^29^. Additionally, use of MRI before and after FUS application can serve the dual purpose of guiding treatment and monitoring for edema or hemorrhage^30^, which is a risk factor associated with intracranial delivery^31^, and to some extent with FUS^32^. The safety of FUS continues to improve as advances are made in understanding BBB permeabilization dynamics in preclinical models and in human patients^33,34^. As preclinical examples, the observation that closure time is unaltered by permeabilization volume^35^, and the development of an acoustic emission-based controller to adjust application pressures^36^, which was used in this study, are examples of these advances. Recent clinical trials demonstrated that single and repeated FUS applications to a variety of brain regions led to controlled, consistent and reversible BBB permeability–with BBB restoration within 24 hours^33,34,37–39^.

In addition to providing a less invasive method for drug delivery to the brain, transcranial MRIgFUS broadens the options for intravenous AAV administration to include serotypes that cannot innately overcome the BBB (reviewed in^40,41^). For serotypes that can overcome the BBB, such as AAV9, gene delivery to specific areas of the brain can be achieved with MRIgFUS by using an intravenous dose 50–100-fold lower than what is normally required to bypass the BBB^8,9,41^. A caveat of using intravenous delivery of AAVs for MRIgFUS delivery to the brain is that a significant amount of the AAV will be lost through biodistribution within peripheral organs, particularly the liver^42^. Additionally, with high rates of liver transduction comes the risk of T-cell mediated destruction of AAV-transduced hepatocytes, irrespective of whether or not transduction results in expression of a transgene^43^. In order to mitigate these challenges, AAV serotype designs should aim to limit peripheral biodistribution, which will also increase the amount of AAV circulating in the bloodstream, and remaining available for delivery to the brain using MRIgFUS.

Here, both AAV1&2 and AAV6 GFP gene copy numbers within the liver were an order of magnitude higher than in the heart, lung, kidney, and skeletal muscle. This is in agreement with the findings of a previous study which reported biodistribution as vector genomes per cell after systemic delivery of AAV1, 2, or 6^44^. The mosaic AAV1&2 was used in this study to combine the high neuronal tropism of AAV2 with the greater brain parenchyma diffusion of AAV1^16^. Previous studies comparing the parental AAV serotypes 1, 2, and 6 have shown no significant difference in biodistribution to the liver between these serotypes^4,44^, which suggests that, in this study, the high amount of AAV1&2 found in the liver could be related to interactions of the mosaic serotype.

To prevent expression of the transgene in the liver and other peripheral organs after transduction by AAV, the syn promoter was utilized. We previously demonstrated that systemic injection of AAV1&2 under control of the constitutive human beta actin (HBA) promoter resulted in GFP expression in a broad variety of cell types within the brain, whereas a glial fibrillary acidic protein (GFAP) promoter limited GFAP expression to astrocytes^45^. Neither the HBA nor GFAP promoter prevented transgene expression in the liver after systemic delivery^45^. As demonstrated in 2003 by Kügler et al., the syn promoter led to neuron-specific GFP expression after intracranial injection of adenovirus to the striatum, corpus callosum, and substantia nigra^46^. This was facilitated via a promoter sequence derived from the human synapsin 1 gene promoter, which is the same syn promoter used in the current work. Here, and in Kügler et al.^46^, the syn promoter successfully constrained GFP expression to neuronal populations in the brain. In 2014, Wang et al. demonstrated that MRIgFUS delivery to the caudate-putamen of AAV1 and AAV2 under the syn promoter resulted in neuronal GFP expression, with approximately 5% of transgene-positive cells identified as NeuN-negative^25^. The phenotype of these NeuN-negative cells remains to be characterized^25^. A limitation to the current study is that it did not evaluate transgene expression in peripheral neurons after systemic injection. Peripheral neuron transduction has been previously demonstrated using AAVs and it remains an important area for future investigation in the development of gene therapies^47–52^.

Compared to intracranial injections of AAVs to the brain, a benefit of MRIgFUS delivery is the surface area that can be covered for gene transfer. Intracranial injection allows for concentrated delivery of AAVs in a brain region, with limited diffusion. AAV2-mediated transgene expression has been reported within approximately 1.1 mm^3^ from intracranial injection sites^53^. To put this in perspective, the human hippocampus (unilateral) is on the order of 2.5 cm^3^ ^54^, suggesting that multiple injection sites would be necessary to supply virus to a significant portion of the hippocampus. The flexibility of MRIgFUS to cover volumes ranging from few mm^3^ to several cm^3^, in a controlled manner to the selected brain area(s), overcomes the challenge of multiple intracranial injections^55,56^. The flexibility in target volumes afforded by MRIgFUS, has made current clinical trials of FUS-mediated BBB opening in neurodegenerative disease possible, where progressively larger volumes of BBB are being opened^34,37–39,57^.

Within the regions of the brain showing GFP-expression, the mean percentages of transgene-positive neurons after 2 weeks were of 2.3% using AAV6-syn-GFP and 1.0% using AAV1&2-syn-GFP. In comparison to intracranial injections, Blits and colleagues conducted a study in which AAV (5 × 10^5^ genome copies) was injected into the red nucleus of rats, leading to transgene expression in approximately 1–10% of cells^20^. Specifically, the percentages of cells expressing the transgene were 1% for AAV1, less than 1% for AAV2, and 10% for AAV6 1 week after injection of AAVs under control the constitutive cytomegalovirus promoter^20^. Under the syn promoter, single-stranded AAV8-syn injected to the striatum resulted in 2–9% of all cells expressing transgene 8–21 days after delivery in mice^58^. Although a direct translation of percentage of transgene-positive cells to functional output is not readily available, a study using a mouse model of lysosome storage disease injected AAV2 (1.5 × 10^5^ infectious units of virus per injection site) expressing β-glucuronidase into the brain, and found a reduction in lysosomal distension, as well as an absence of pathological ganglioside accumulation^59^. The percentage of cells expressing transgene was not reported in the latter study, but comparison with the publication from Blits and colleagues using a similar injected dose of AAV2 suggests that less than 1% of cells were transgene positive. Another study using a higher dosage of AAV2 (1 × 10^8^ VG) to express neurotrophic growth factor in the basal forebrain in aged rats found a significant increase in the number of cholinergic neurons^60^. Similarly, Richichi and colleagues show that intracranial infusion of AAV2 or AAV1/2 expressing neuropeptide Y at 1.06 viral particles into the rat hippocampus led to a 50–70% reduction in EEG-measured seizures^61^. In a Parkinson’s disease trial, AAV2 expressing glutamic acid decarboxylase injected into the subthalamic nuclei at 4.5 × 10^10^ VG bilaterally demonstrated improvement in the Unified Parkinson’s Disease Rating Scale^62^; though this is difficult to compare with the present study as cell transduction efficiency may differ between humans and rodent models. Lastly, Hsu and colleagues have described MRIgFUS delivery of AAV2 under a constitutive promoter that reached 40% transduction efficiency in astrocytes and 12% transduction efficiency in neurons 3 weeks after delivery within selected 100 × 100 µm^2^ regions of the full 2 mm × 10 mm FUS spot^7^. Together, these studies suggest that even 1–2% of cells expressing transgene in the total focal area could provide a functional outcome, contingent on delivery location, transgene selection and optimal dosage.

The present study investigated MRIgFUS gene delivery to the hippocampus and striatum, regions relevant to neurodegenerative disorders. The hippocampal formation, heavily implicated in Alzheimer’s disease^26^, is a brain area where adult neurogenesis occurs^63^. FUS applications to the hippocampus have been shown to increase the number of proliferating cells in the subgranular layer of the dentate gyrus, as well as the number of immature and mature neurons, overall promoting hippocampal neurogenesis^63–65^. In addition, FUS applications to the brain in mouse models of Alzheimer’s disease have been shown to activate glial cells, reduce amyloid and tau pathology, and improve cognitive functions^64,66–68^. Taken together, FUS-mediated gene delivery to the hippocampus could be used to harness the benefits of neuronal^63–65^ and glial^66,67^ modulation, in addition to supplying a therapeutic transgene to affected brain areas. Our work begins to address gene expression and biodistribution in off-target organs, specifically the sequestering of virus in the liver after intravenous delivery, by exploring AAV serotype and cell-specific promoter combinations.

## Conclusion

FUS-mediated AAV-syn-GFP delivery to the brain allowed for non-surgical, neuron-specific gene expression in the targeted regions of interest, while minimizing expression in the liver after intravenous injection of AAV. Compared to AAV9 and AAV1&2, AAV6 resulted in significantly lower biodistribution within the liver, and a comparable number of neuronal cells expressing the transgene in the brain after FUS application compared to AAV1&2. AAV is a particularly viable choice for MRIgFUS-mediated gene delivery to the CNS, provided the low immunogenicity, safety, and ability of AAVs in general to transduce non-replicating cells and result in long-term expression of transgenes^69^.

## Methods

### Animal preparation

C57BL/6 mice (Jackson Laboratories, Bar Harbor, ME) were used at 13 weeks of age, with an average weight of 20 g per mouse. All animal procedures were carried out in compliance with the Canadian Council on Animal Care and the Animals for Research Act of Ontario guidelines, and with the approval of the Sunnybrook Research Institute Animal Care Committee.

### Virus preparation

Single stranded AAV6, mosaic AAV1&2 and AAV9 expressing GFP under control of the syn promoter, and AAV1&2 expressing GFP under control of the human beta actin (HBA) promoter were prepared as previously described^18,70^. Rep and cap genes of the AAV wildtype genome were replaced with transgenic sequences as previously described^71^. AAV1&2-syn-GFP and AAV6-syn-GFP was delivered via tail vein catheter at a dose of 3 × 10^9^ vector genomes/gram (VG/g).

### Magnetic resonance imaging-guided focused ultrasound (MRIgFUS)

Mice were anesthetized via inhalation of isofluorane. The hair from the head and neck was removed with a depilatory cream. A 26-G angiocatheter was inserted into the tail vein to facilitate intravenous injections. The mice were placed in dorsal recumbency over an MRI radiofrequency surface coil as previously described^72^. Procedures of MRIgFUS were carried as described below and, after recovering from anesthesia, mice were returned to their cages.

MRI images of the brain were generated using a 7 T MRI (Bruker BioSpin MRI GmbH, Ettlingen, Germany). T2-weighted images were used to select regions of interest (Fig. 6a). Definity ultrasound contrast microbubbles (Lantheus Medical Imaging, North Billerica, MA, USA; 0.02 ml/kg), followed by saline (200 µl) were injected through the tail vein catheter, at the start of the FUS application. Ultrasound was generated using a 1.68 MHz spherically focused transducer (aperture: 7 cm, F-number: 0.8), driven using a radio frequency power amplifier and function generator. Focused ultrasound sonications consisted of 10 ms bursts, at a 1 Hz burst repetition frequency, for a duration of 120 s. A 4.8 mm diameter wideband polyvinylidene fluoride hydrophone was used to monitor acoustic emissions, which were used to control the acoustic exposures, as described previously^36^. For all sonications, the acoustic pressure amplitude level was increased in a step-wise manner while recording the sub-harmonic emissions. When a sub-harmonic (840 kHz) signal was detected, the pressure amplitude was reduced to 50% of the detection level and used for the remaining duration of the sonication. This allowed for the unique vascular composition within each focal spot to receive the appropriate acoustic emission pressure for BBB opening. Thereafter, virus injection (3 × 10^9^ VG/g) was followed by saline (200 µl), and Gadodiamide MRI contrast agent (Omniscan, GE Healthcare Canada, Mississauga, ON, Canada, 0.2 ml/kg) and saline again (200 µl). Two FUS spots were aimed at the hippocampus, and one at the striatum (Fig. 6b–e) with a resolution of 1–2 mm. Post FUS application, contrast-enhanced T1-weighted MRI images were taken to confirm BBB permeability, as indicated by areas of enhancement (Fig. 6b, arrows). This targeting approach also covered regions of the cortex and thalamus (Fig. 6d,e). To be as rigorous as possible, data from all regions targeted are reported, rather than only the hippocampus and striatum.

### MRI Enhancement

Enhancement at individual FUS spots was quantified from an average of a 3X3 pixel region of the post-FUS T1-weighted MRI image (Fig. 6b) and expressed as a percentage increase from background enhancement using Matlab (MathWorks, Natick, MA, USA). A two-tailed, unpaired t-test was used to compare the enhancement of all FUS-targeted spots in the AAV1&2-syn-GFP versus AAV6-syn-GFP groups (n = 9 FUS spots, as indicated by areas of enhancement on the post-FUS MRI; 3 spots per animal, from 3 animals per experimental group), and between FUS spots which included regions of the hippocampus (n = 5 per group), or regions of the striatum (n = 4 per group) (Fig. 5a–c) (GraphPad Prism 5, GraphPad Software, La Jolla, CA, USA). The key biological driver of AAV biodistribution, even within the same mouse, is the variable degree of BBB opening^73,74^, which occurs independently in each spot. Therefore, each FUS spot was considered as a separate evaluation.

### Histological processing

Two weeks following MRIgFUS, the animals were anesthetized using an intraperitoneal injection of ketamine (75 mg/kg) and xylazine (10 mg/kg). Transcardial perfusion was then performed using 0.9% saline and 4% paraformaldehyde solution in 0.1 M PO_4_. The brain, as well as the lung, heart, liver, spleen, kidney, and quadriceps muscle (peripheral organs) were collected and post-fixed for 24 h in 4% paraformaldehyde solution. Thereafter, tissue was stored at 4 °C in 30% sucrose solution. Brains and peripheral organs were mounted in Tissue-Tek OCT (Sakura, Torrance, CA, USA) and frozen with dry ice on a sliding microtome for coronal sectioning at 40 μm thickness. Sections were stored in cryoprotective glycerol solution at − 20 °C.

### Immunohistochemistry

40 μm-thick, free floating sections were washed three times for 5 min in phosphate-buffered saline (PBS, pH 7.4) before incubation in blocking solution (PBS + +). Blocking solution consisted of 10% donkey serum (Wisent Bioproducts, Saint-Jean Baptiste, QC, Canada), and 1% Triton X-100 (Sigma-Aldrich Canada, Oakville, ON, Canada). Brain sections were incubated at 4 °C for 72 h in rabbit anti-GFP (1:500; Millipore, AB3080, Bedford, MA, USA), goat anti-glial fibrillary acidic protein (GFAP) (1:500, Santa Cruz, SC-6170, Dallas, TX, USA), and guinea pig anti-neuronal nuclear antigen (NeuN) (1:500, Millipore ABN90) in blocking solution. Sections were then washed in PBS three times for 5 min (washing sequence) and incubated in PBS +  + with donkey anti-rabbit biotin (1:80, Jackson ImmunoResearch, 711–065-152, West Grove, PA, USA), donkey anti-goat Cy3 (1:200; Jackson ImmunoResearch, 705–166-147), and donkey anti-guinea pig Cy 5 (1:200; Jackson ImmunoResearch, 706–175-148) at room temperature for 2 h. After an additional washing sequence the sections were incubated in PBS +  + with Alexa Fluor® 488-conjugated streptavidin (1:200, Jackson Immunoresearch, 016–540-084) at room temperature for 1 h. After a washing sequence the sections were exposed to 4′,6-Diamidine-2′-phenylindole dihydrochloride (DAPI) nucleic acid counterstain (1:10,000, Invitrogen, D3571, Eugene, OR, USA) in PBS for 5 min, and washed again before mounting on a microscope slide with polyvinyl alcohol (Sigma-Aldrich, St Louis, MO, USA) and 1,4 diazabicyclo(2.2.2)octane (Sigma-Aldrich) (PVA-DABCO) and a coverslip.

Liver, kidney, muscle, heart, spleen, and lung sections were treated as described above, using the anti-GFP primary antibody, anti-rabbit biotin and Alexa Fluor® 488-conjugated streptavidin secondary antibodies, and DAPI.

### Imaging

Images were captured using an apochromatically corrected 20 × objective (NA 0.75) (Fig. 3c–f, h–k) and 60 × objective (NA 1.4) (Fig. 3g,l) on a Nikon A1 laser scanning confocal microscope (Nikon Instruments, Melville, NY, USA). Images (Fig. 3c–f, h–k) are presented as projections of 17, 1.6 μm Z-stacks. Virtual slice images (Figs. 3a,b, 7a–c) were obtained using a 20 × objective (NA 0.5) on an AxioImager M2 (Carl Zeiss, Toronto, ON, Canada). Adjacent images were assembled into a single mosaic using Stereo Investigator software (MBF, Biosciences, Williston, VT, USA) (Figs. 3a,b, 7a,b). The 3D reconstruction image was generated using 10, virtual slice z-stack images and BrainMaker software (MBF, Biosciences, Williston, VT, USA) (Fig. 7c).

### Cell counting

Ten, 40 μm-thick sections from each FUS spot were used at an interval of 1 in 2 sections for quantification. Distinct, non-overlapping areas of GFP expression, corresponding to locations of enhancement seen on the post-FUS MRI of each animal, were used to delineate regions of interest for each brain region (i.e. striatum, hippocampus, cortex, and thalamus) (Fig. 6). Within these regions of interest, the total numbers of GFP and NeuN-positive cells were quantified and expressed as the percentage of NeuN-positive neurons expressing GFP (Fig. 4). Quantification was done using the optical fractionator probe (Stereo Investigator, MBF, Biosciences, Williston, VT, USA) at 63x (oil objective; NA = 1.4) with systematic random sampling on a Zeiss AxioImager M2 microscope (Carl Zeiss, Toronto, ON, Canada). The average coefficient of error (Gundersen m = 1) was 0.09 for GFP/NeuN-positive cells, and 0.02 for GFP-negative/NeuN-positive cells. GFP-positive/NeuN-negative cells were not observed. The percentage of GFP-positive cells were calculated from 9 focal spots and reported as an average from n = 3 mice per AAV group (3 focal spots per animal). For each FUS spot the Pearson correlation between enhancement and number of transgene-positive cells in the corresponding region of GFP expression was measured to evaluate an effect of MRI enhancement on general AAV transgene expression (n = 18, 9 from each AAV experimental group) (Graph Pad Prism 5, GraphPad Software) (Fig. 4f). Since a significant (*p* = 0.023, Pearson r = 0.531) correlation between number of GFP-positive cells and MRI enhancement was found, the mean percentage of GFP-positive cells was compared between AAV serotype groups using an analysis of covariance (ANCOVA) with MRI enhancement as the covariate (SPSS, Chicago, USA). This allows for analysis of AAV serotype on percentage of GFP-positive cells to be isolated from variances in BBB opening (as measured by MRI enhancement). Bonferroni’s post-hoc analysis was used to determine *p* values, and a value of less than 0.05 was considered statistically significant.

### DNA extraction

Peripheral organ sections were processed for DNA extraction using chloroform and phenol as described previously^75^. Extraction samples were then quantified with a Nanodrop (Thermo Scientific, Waltham, MA, USA). For some samples the phenol–chloroform extraction method yielded an insufficient concentration of DNA, in which case additional tissue was processed using a QIAamp® DNA FFPE Tissue Kit (Qiagen Cat. 56,404, Hilden, Germany).

### Droplet digital PCR

QX200 droplet digital PCR (ddPCR) system (Bio-Rad) was used to quantify the number of GFP gene copies per cell in peripheral tissue, normalized to the ApoB gene. The GFP gene was selected as a target since this is a unique and consistent variable between the three serotypes tested, and only present as one copy per viral genome. For GFP detection a forward primer 5′-ACTACAACAGCCACAACGTCTATATCA-3′, reverse primer 5′-GGCGGATCTTGAAGTTCACC-3′ (Invitrogen), and probe 5′-6-FAM-CCGACAAGC-ZEN-AGAAGAACGGCATCA-Iowa Black FQ-3′ (Integrated DNA Technologies, Coralville, IA, USA) sequences were used. The ApoB forward primer 5′-CGTGGGCTCCAGCATTCTA-3′, reverse primer 5′-TCACCAGTCATTTCTGCCTT TG-3′, and probe 5′-HEX-CCTTGAGCA-ZEN-GTGCCCGACCATTC- Iowa Black FQ-3′ (Integrated DNA Technologies) sequences were used. PCR was performed in a 20 µl volume containing 10 ng of genomic DNA, 900 nM of the forward and reverse GFP primers, 250 nM GFP probe, 900 nM of the forward and reverse Apob primers, 250 nM Apob probe, and 10 µl of 2X ddPCR supermix for probes (Bio-Rad). Each ddPCR assay mixture was loaded into a disposable droplet generator cartridge (Bio-Rad). Then, 70 µL of droplet generation oil for probes (Bio-Rad) was loaded into each of the eight oil wells. The cartridge was then placed inside the QX200 droplet generator (Bio-Rad). When droplet generation was completed, the droplets were transferred to a 96-well PCR plate (Eppendorf) using multichannel pipet. The plate was heat-sealed with foil and placed in C1000 Touch Thermal Cycler (Bio-Rad). Thermal cycling conditions were as follows: 95 °C for 10 min, then 44 cycles of 94 °C for 30 s and 60 °C for 1 min, and 98 °C for 10 min, and a 4 °C indefinite hold. FAM fluorescent signal, labeling the GFP DNA sequence, and HEX fluorescent signal, labeling the ApoB gene sequence in each droplet were counted using a QX200 digital droplet reader, and analyzed by QuantaSoft analysis software ver.1.7.4.0917 (Bio-Rad). The mean GFP gene copy number per cell, between the AAV9, AAV6 and AAV1&2 experimental groups, was evaluated using an ANOVA and Bonferroni’s post-hoc analysis. In contrast to the other peripheral organ biodistribution data, the spleen and lung results were significant for both the Levene’s test for homogeneity of variance and the Shapiro–Wilk test for normal distribution, and were therefore analyzed using a nonparametric pairwise Mood’s median test (SPSS, IBM, Armonk, NY and GraphPad Prism 8, GraphPad, San Diego, CA).

## Data Availability

The datasets generated during and/or analysed during the current study are available from the corresponding author on reasonable request.
